# The Effects of Extended-Access Cocaine Self-Administration on Working Memory Performance, Reversal Learning and Incubation of Cocaine-Seeking in Adult Male Rats

**DOI:** 10.13188/2330-2178.1000035

**Published:** 2017-04-27

**Authors:** Christina Gobin, Marek Schwendt

**Affiliations:** Department of Psychology, College of Liberal Arts & Sciences University of Florida, Gainesville, USA

**Keywords:** Cocaine, Working memory, Reversal learning, Relapse, Cocaine-seeking

## Abstract

Cocaine use disorder is characterized not only by the high rate of relapse, but also by deficits in cognition and prefrontal cortical function. Still, the relationship between cognitive impairment and cocaine-seeking remains poorly understood. The current study used a rodent model to determine the effects of extended access cocaine self-administration on cognitive performance in a prefrontal cortex-dependent delayed match-to-sample/non-match-to-sample (DMS/DNMS) task. Further, this study sought to investigate how post-cocaine changes in cognitive performance correlate with cue/context-induced cocaine-seeking following a prolonged period of abstinence. Animals were trained to self-administer cocaine during 6 daily 1 hour-long sessions followed by 12 days of extended, 6 hour-long access. The extended access cocaine rats exhibited robust self-administration behavior and escalation of cocaine intake. Next, DMS/DNMS task was used to evaluate working memory capacity and reversal learning performance over a range of 0 – 30 s delays. Although this study failed to detect a major cognitive impairment, extended access to cocaine resulted in the persistent working memory/DMS deficit at a moderate cognitive load (10 s delay). There were no changes in the reversal learning/DNMS performance. It is likely that the parameters of the DMS/DNMS task, as used in the current study, exceeded acquisition capacity of rats thus obscuring cocaine effects at longer delays. Finally, rats showed a robust relapse of context/cue-elicited cocaine-seeking following the 45 - day abstinence. However, the intensity of cocaine-seeking did not correlate with the deficit in the DMS task. In conclusion, future studies must re-evaluate whether a more robust relationship between post-cocaine cognitive performance and cocaine-seeking can be detected under adjusted DMS/DNMS conditions.

## Introduction

Cocaine addiction (cocaine use disorder) is a chronic relapsing disorder, characterized by compulsive drug seeking and decreased ability to limit intake [1]. Loss of control over drug-taking has been (partially) attributed to a dysfunction of the prefrontal cortex (PFC) [2]. In support, decreased activity of the PFC (also termed hypofrontality) has been documented in subjects with cocaine use disorder using functional imaging [3]. Coinciding with hypofrontality is the emergence of cognitive impairments mainly related to attention, working memory, impulsivity and cognitive flexibility [4–7]. Critically, these impairments can already be detected shortly after discontinuation of cocaine use and persist over an extended period of abstinence [8]. For example, one study found significant impairments in cocaine addicts versus healthy controls on measures of attention, executive functioning, and global cognitive impairment after six weeks of abstinence that persisted even after six months of abstinence [9]. As cocaine-induced cognitive impairments not only negatively affect well-being of affected individuals, but also increase the risk of relapse and worsen the treatment outcomes, development of relevant animal models and characterization of these deficits is of high clinical importance [10–12].

To this date, only a handful of animal studies explored post-cocaine cognitive deficits using a validated model of rat self-administration [6,13–15]. For the most part, these studies are in agreement that cocaine self-administration in rats produces deficits in spatial working memory. In addition, a subset of studies supports the idea that extended (not limited) access to self-administered cocaine is more likely to produce persistent motivational and cognitive deficits, such as impaired working memory and increased (incubated) cocaine-seeking [6,13,16]. Critically, while human studies suggest that post-cocaine cognitive deficits often reliably predict increased likelihood of relapse, this has not been thoroughly investigated in animals [10–12]. This knowledge gap motivated the current study to explore the relationship between post-cocaine working memory and reversal learning deficits and persistent cocaine-seeking.

In this study, we evaluated the performance in a PFC-dependent, operant delayed match-to-sample (DMS) / non match-to-sample (DNMS) task (see [17]) in animals with a history of extended cocaine self-administration. Further, to investigate the link between PFC-dependent cognitive dysfunction and drug-seeking, animals underwent (cue + context)-induced reinstatement, following the completion of the DMS/DNMS testing. We hypothesized that a history of extended access to cocaine would decrease working memory performance, impede reversal learning and that these cognitive impairments would correlate with increased (incubated) cocaine-seeking after protracted, 45 day-long abstinence.

## Materials and Methods

### Animals

Adult male Sprague Dawley rats (Charles River; 275 g on arrival; n = 16) were acclimated to the animal facility for one week prior to surgeries. They were housed individually and maintained on a 12 h light/dark cycle (lights off at 0700). Testing took place during their dark cycle. Food and water were available ad libitum prior to surgery. All animal procedures were approved by the Institutional Animal Care and Use Committee of the University of Florida (IACUC) and were performed in accordance with the Guide for the Care and Use of Laboratory Animals. Two rats from the saline group were excluded from all analyses because they were detected as outliers (in either the relapse test or the DMS task) using the Grubb’s test.

### Cocaine self-administration

All rats underwent surgical jugular catheter implantation as described previously [18]. After surgery, all rats were allowed one week to recover in their home cages with food and water ad libitum until the day before behavioral testing when they were food restricted 85% of their body weight. Catheters were flushed daily with 0.1 ml of heparinized saline. Following recovery, rats were randomly assigned to either cocaine or saline groups and underwent self-administration procedures using modified operant chambers (30 × 24 × 30 cm; Med Associates, St. Albans, VT) equipped with two standard rat nose pokes. The standard operant chambers in the self-administration portion of the experiment were modified (mesh wire floor, striped wall pattern, vanilla scent) in order to provide a context that was distinct from the one used for the operant DMS task. Animals assigned to the cocaine group received response-(active nose poke)-contingent infusions of cocaine (0.35 mg/100 μL infusion; provided by the National Institute on Drug Abuse, Bethesda, MD) on a FR1 schedule of reinforcement. Each infusion was paired with a 5 s presentation of a light + tone cue followed by a 20 - second ‘time-out’ period. During the time-out period, the cue light remained illuminated, while nose-poking was recorded but not reinforced. Inactive nose poke responses were not reinforced, but were recorded. Rats in the cocaine group underwent 6 daily 1 hr (short access, ShA) sessions, followed by 12 daily 6 hr (long access, LgA) sessions. Rats assigned to the saline group, underwent 18 daily 2 hr sessions, during which they received 30 programmed infusions of saline paired with a 5 s presentation of a light + tone cue. This was done in order to provide a sensory and contextual experience similar to that of cocaine rats. After the conclusion of self-administration, animals underwent 45 days of abstinence. Studies have shown that this extended time window accompanies persistent cognitive deficits, as well as an incubation of craving following chronic cocaine abuse [6,16]. Animals underwent training and testing in the operant DMS/DNMS cognitive task during this abstinent period.

### Operant delayed match-to-sample/non-match-to-sample task

**Figure 1:**
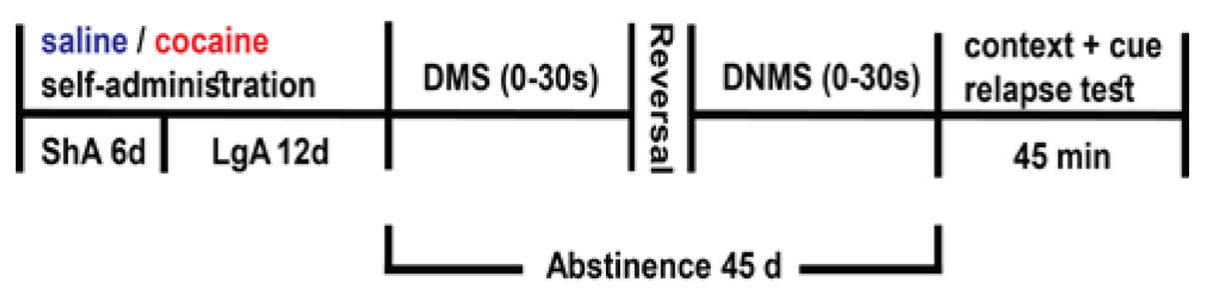
Timeline of the study. Relapse test was conducted on day 45. DMS: Delayed Match-to-Sample; DNMS: Delayed Non-Match-to-Sample; LgA: Long Access; ShA: Short Access

Delayed match-to-sample (DMS) and non-match-to-sample (DNMS) tasks are based on previously published procedures [17]. Each session was conducted daily for 40 minutes. Every trial was composed of three components - a sample component, a delay period and a choice component. In the sample component, a left or right lever was chosen at random by the computer such that each lever was presented approximately the same number of times in each session. Pressing the sample lever resulted in retraction of that lever, delivery of a sucrose pellet, and activation of the delay timer. After the delay interval, the choice phase was initiated wherein both levers were extended. A correct response of choosing the same side lever presented in the sample phase resulted in delivery of a sucrose pellet. An incorrect response, on the other hand, resulted in a 6 s time-out period wherein no sucrose pellet was delivered, the house light was extinguished, and both levers were retracted. Rats were trained without any delays between the sample and choice component prior to being trained at three delay sets. Rats were required to reach a criterion for two consecutive days at each delay set. The criterion was ≥ 80% correct during initial training without delays {0 s}, 70% at the {0 s, 10 s} delay set, and 60% at {0 s, 10 s, 20 s} and at {0 s, 10 s, 20 s, 30 s}. Criterion was applied on overall responding rate across each delay set. In the testing phase, rats were tested on their performance at the interval set {0 s, 10 s, 20 s, 30 s} for a block of five days. Next, rats were tested for another block of five days in the non-match-to-sample task wherein the rule was switched at the same final delay set {0 s, 10 s, 20 s, 30 s}. The switched rule in the non-match-to-sample task required the rat to choose the lever opposite from the sample lever for a sucrose reward (Figure 1).

### Relapse test

On day 45 following the last self-administration session, rats were reintroduced to the drug context for a 45 minute-long relapse test (Figure 1). Nose pokes within the active holes generated light and tone cues, but infusions of cocaine were no longer administered. In the relapse test for the control saline group, each rat received a light and tone cue every 4 minutes, but no infusions of saline were administered.

### Statistical Analyses

IBM SPSS Statistics (Version 21) software was used to analyze all data, and all figures were graphed using GraphPad Prism (Version 5.03) software. The alpha level was set at p < 0.05 for all statistical analyses used. Self-administration data was analyzed with repeated measures ANOVA to compare nose pokes in the active hole versus inactive hole across days throughout the LgA sessions. A repeated measures ANOVA with Greenhouse - Geisser correction was used to analyze the average number of cocaine infusions throughout the 18 cocaine self-administration sessions. In addition, a paired sample t-test was used to compare the total cocaine intake (mg/kg) between the two 6 - day blocks of the LgA sessions.

For the DMS and DNMS tasks, a one-way ANOVA was used to assess group differences in the number of days spent in training before entering the five day testing period. Percent correct was the primary dependent measure in the DMS and DNMS tasks. Percent correct at each delay interval {0 s, 10 s, 20 s, 30 s} was averaged over the five day testing block for a measure of the total percent correct at each delay. A repeated measures ANOVA was conducted to assess group differences in total percent correct at each delay. In order to evaluate and interpret behavior in these tasks, percent correct at each delay interval needs to be above chance to indicate that the rats’ responding is not due to chance but rather to successful acquisition of the task rules. To demonstrate acquisition for each group, the group mean at each delay interval was compared to a hypothetical mean of 50% using a one-sample t-test. Only delays that were greater than this hypothetical mean were included for further analysis. Repeated measures ANOVAs were conducted to assess group differences in mean total percentage correct at a particular delay (in which group performance was above chance) across the five day testing block.

For relapse data, a one-way ANOVA was conducted to compare the saline and cocaine groups on the number of nose pokes in the active hole during a 45 minute relapse test. Finally, to explore the relationship between cognitive performance and relapse, Pearson correlations were conducted to compare percent correct with number of nose pokes in the active hole.

## Results

### Extended daily access to cocaine produces robust self-administration behavior and escalation of cocaine intake

**Figure 2:**
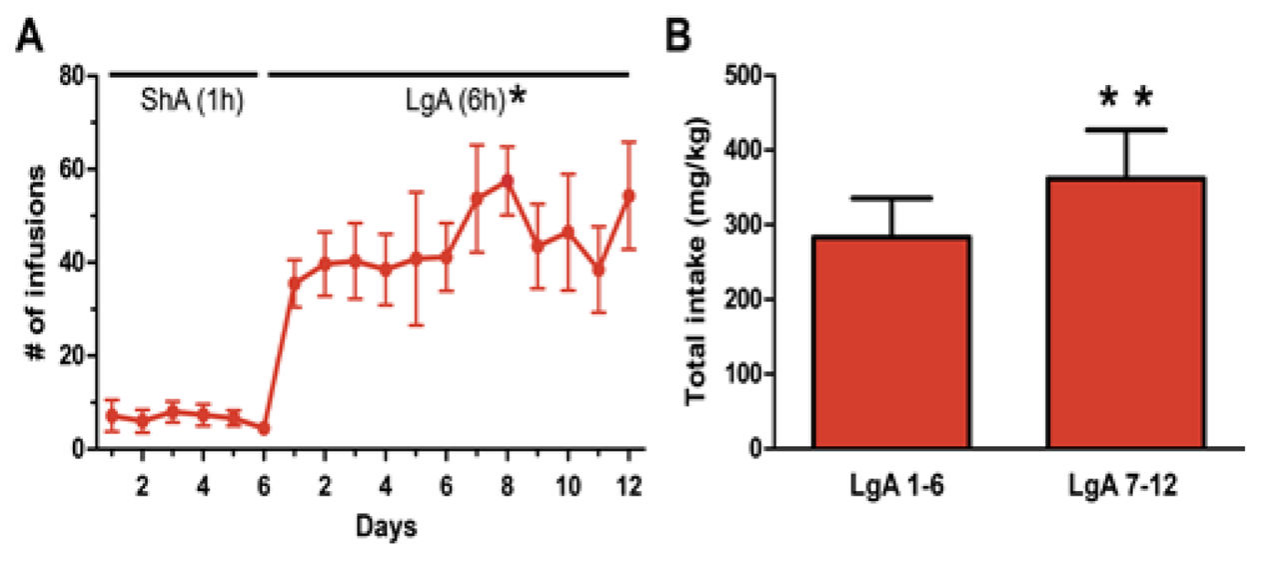
Cocaine self-administration and intake. **(A)** Mean number of cocaine infusions during short-access (1 hr) and longaccess (6 hrs) self-administration. Each day of long access is significantly different from the last day of short access self-administration (*p < 0.05). **(B)** Total cocaine intake (mg/kg) during the first six days and the last six days of long-access self-administration. Mean total cocaine intake during the last six day block of testing is significantly greater than the first six day block of testing. (*p < 0.05= LgA vs. ShA; **p < 0.01 = LgA 7–12 vs. LgA 1 – 6). LgA: Long Access; ShA; Short Access; n = 6 – 8.

All animals self-administered cocaine, or received saline infusions, during 6 daily short-access (ShA, 1 hr) and 12 daily extended-/long-access (LgA, 6 hr) sessions (Figure 1). Cocaine animals successfully acquired self-administration procedure, indicated by discrimination between active vs. inactive nose pokes throughout the experiment (*F* (1, 5) = 9.28, *p* < 0.05; data not shown). When given extended (LgA) access to cocaine, animals displayed a robust self-administration behavior as evidenced by an increase in average number of daily infusions (*F* (2.4, 11.8) = 11.8, *p* < 0.05) when compared to ShA (Figure 2A). In addition, LgA animals escalated cocaine self-administration as evidenced by an increase in total cocaine intake from the first six days of LgA (LgA 1 – 6) to the last six days (LgA 7 – 12; t (1, 5) = 4.015, *p* < 0.01; Figure 2B). The mean cumulative cocaine intake over 18 days of self-administration was 690.9 ± 122 mg/kg.

### Cocaine and saline animals did not differ in DMS and DNMS performance at each of the delays

**Figure 3:**
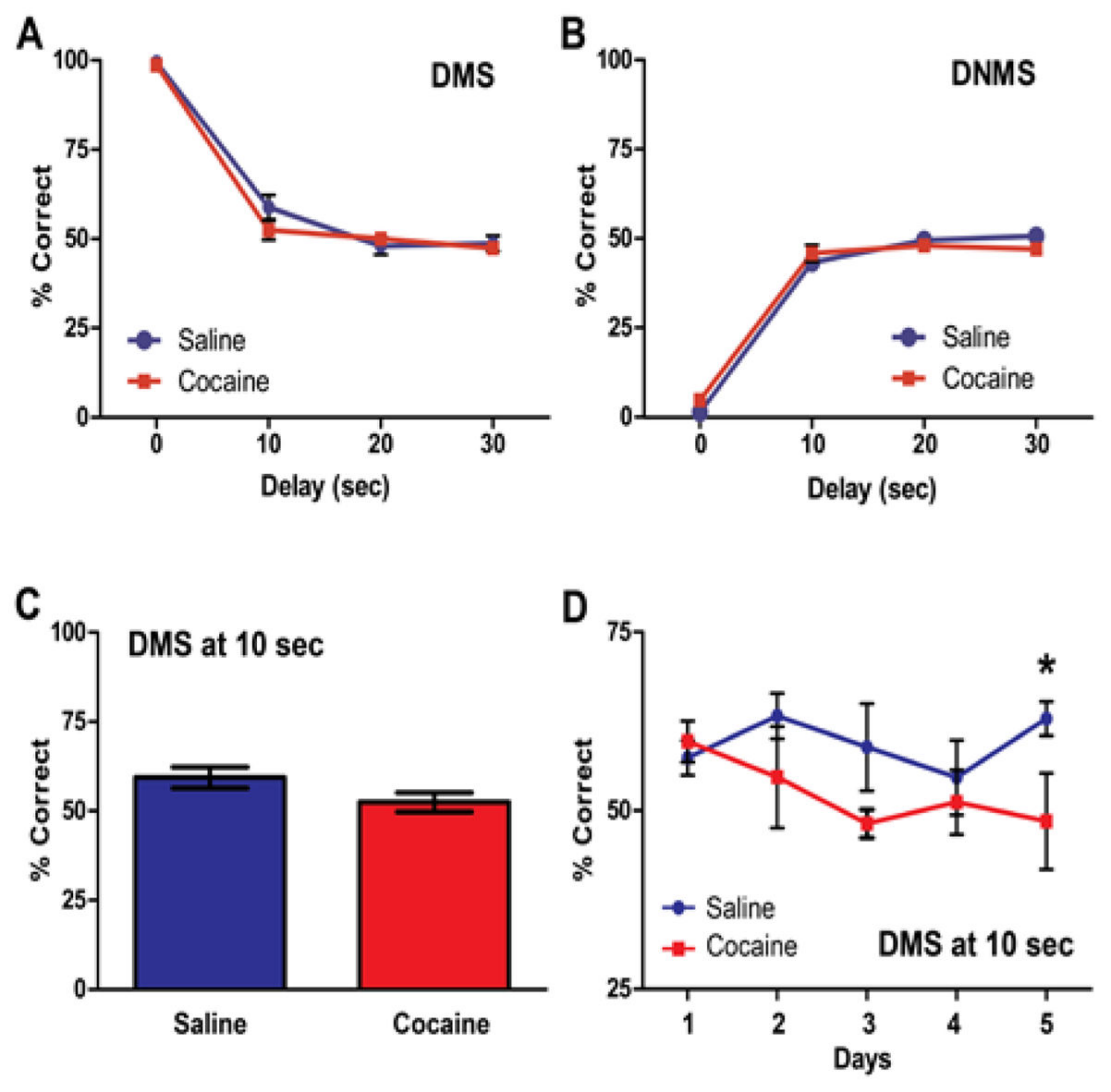
Performance in the DMS and DNMS tasks. **(A)** Percent correct at each delay interval averaged over the five day testing block during the DMS task. There is no significant difference between rats with a history of chronic cocaine and saline rats at each delay interval ((n.s.). **(B)** Percent correct at each delay interval averaged over the five day testing block during the DNMS task. There is no significant difference between rats with a history of chronic cocaine and saline rats at each delay interval (n.s.). **(C)** Percent correct at the 10 s delay averaged over the five day testing block. There is no significant difference between rats with a history of chronic cocaine and saline rats at the 10s delay (n.s.). **(D)** Percent correct on each day of testing at the 10 s delay. Compared to saline rats, rats with a history of chronic cocaine show significant impairment at the 10 s delay on the last day of testing (*p < 0.05 = Coc (d5) vs. Sal (d5)). DMS: Delayed Match-to-Sample; DNMS: Delayed Non-Match-to-Sample; n = 6 – 8.

Following the completion of the self-administration regimen, animals underwent training and testing in DMS and DNMS tasks. The two groups did not differ in the number of days in training before they started the five day testing block (*F* (1, 13) = 0.45; n.s.). The mean percentage correct in the DMS task did not differ significantly between the saline and cocaine groups across the delays {0 s, 10 s, 20 s, 30 s} (*F* (3, 36) = 1.49; n.s.; Figure 3A). An effect of delay was observed (*F* (3, 36) = 275.65, *p* < 0.01). Pairwise comparisons with a least significant difference adjustment showed that average percentage correct at 0 s (M = 99.01, SEM = 0.37) differed significantly from the responding at 10 s (M = 55.89, SEM = 20.09), 20 s (M = 49.41, SEM = 1.83) and 30 s delays (M = 49.21, SEM = 1.33) (p < 0.01). Mean percentage correct at 10 s also differed significantly from the 20 s (*p*< 0.05) and 30 s delay (*p* < 0.01).

The mean percentage correct in the DNMS task did not differ significantly between the saline and cocaine groups across the delays {0 s, 10 s, 20 s, 30 s} (*F* (3, 36) = 2.15; n.s.; Figure 3B). An effect of delay was found (*F* (3, 36) = 413.3, *p* < 0.01). Pairwise comparisons with a least significant difference adjustment showed that average percent correct at 0 s (M = 30.06, SEM = 0.71) differed significantly from the responding at 10 s (M = 45.3, SEM = 1.47), 20 s (M = 48.94, SEM = 10.05), and 30 s delays (M = 49.00, SEM = .98) (*p* < 0.01). Mean percentage correct at 10 s also differed significantly from the 20 s (*p* < 0.05).

### Cocaine and saline animals differ in DMS performance at the 10 s delay on the last day of testing

A one-sample t-test was conducted on all delays to determine if percent correct responding was different from chance (50% correct responses). This analysis revealed that rats of either group showed above chance responding only at 0 s (t (26) = 134.3, *p* < 0.001) and 10 s delays (t (26) = 2.90, *p* < 0.01) in the DMS task. The rats did not display correct, above chance responding at any delay interval in the DNMS task. Thus, further (day-by-day) analysis of behavior was limited to these delays within the DMS condition. The mean percentage of correct responses at the 0s delay did not significantly differ between the saline and cocaine groups across the five day testing block in DMS (*F* (4, 48) = 5.66, *n.s.*; data not shown). Similarly, one-way ANOVA analysis of overall percent correct at the 10 s delay did not show a significant difference between the groups (*F* (1, 13) = 2.74, *n.s.*; Figure 3C) and there was not a significant day by group interaction of mean percentage correct at the 10 s delay in the DMS task (*F* (4, 48) = 1.36, *n.s.*). However, a one-way ANOVA showed a significant difference between the groups at the 10 s delay on the last day (day 5) of testing (*F* (1, 13) = 50.08, *p* < 0.05. The saline rats (M = 62.88, SEM = 2.39) performed better than the cocaine rats (M = 48.50, SEM = 6.72) at this delay on this day. (Figure 3D)

### Cocaine rats display (cue + context)-elicited drug-seeking on post-cocaine day 45

**Figure 4:**
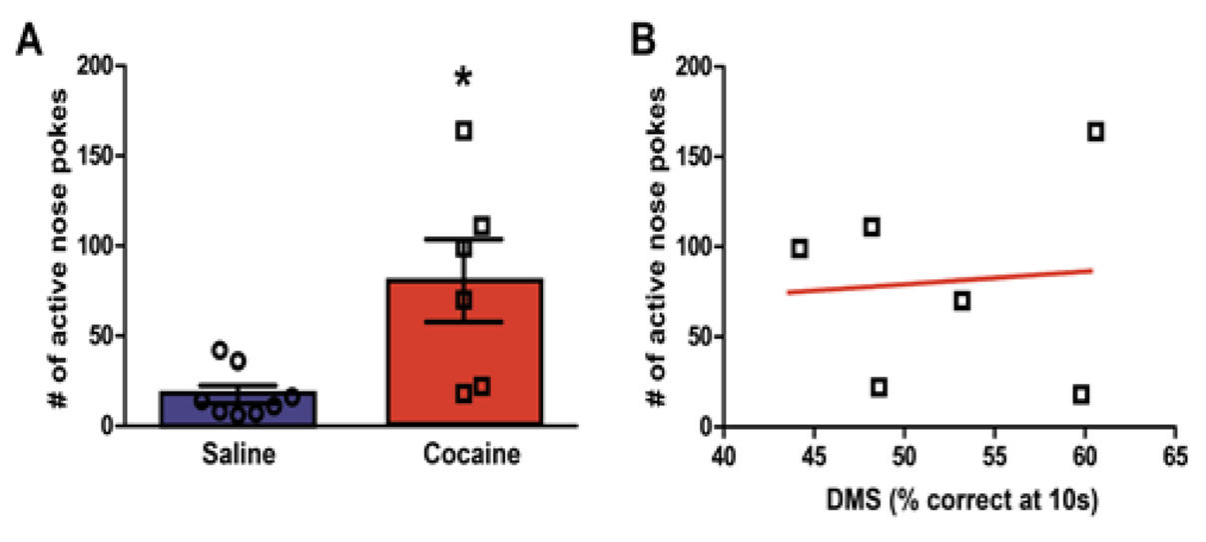
Cue + context relapse to cocaine seeking. **(A)** Average number of nose pokes in the active hole during the 45 minute context + cue relapse test. Compared to saline rats, rats with a history of chronic cocaine demonstrate robust drug seeking during the relapse session, as indicated by significantly greater responding in the previously active hole (*p < 0.05 = Coc vs. Sal). **(B)** Correlation between average percent correct at the 10 s delay with the average number of nose pokes in the active hole for rats with a history of chronic cocaine during the relapse test. There was not a significant correlation between working memory and drug seeking (n.s.). DMS: Delayed Match-to- Sample; n = 6 – 8.

Forty five days following the last self-administration session, animals were re-exposed to the drug-associated context (self-administration chamber with nose pokes and cues available) for the 45 minute relapse test. One-way ANOVA analysis of nose-poking revealed that rats with a history of cocaine self-administration showed significantly higher number of active nose pokes when compared to yoked saline animals (F (1, 13) = 9.65, p < 0.01; Figure 4A).

### A lack of correlation between drug-seeking and cognitive performance in DMS/DNMS task

A Pearson correlation was computed to assess the relationship between the number of nose pokes in the active hole during relapse with the total percent correct at the 10 s delay in the DMS task. There was no correlation between the two variables (r = 0.08, n = 6, n.s.) as shown on Figure 4B. Since significance was found between the saline and cocaine groups at the 10 s delay on the fifth (last) day of testing, a Pearson correlation was also conducted to assess the relationship between the number of nose pokes in the active hole during relapse with the percent correct at the 10 s delay on the fifth day of testing. There was no correlation between the two variables (r = 0.33, n = 6, n.s.; data not shown).

## Discussion

This study sought to examine the effect of extended access cocaine self-administration on working memory performance and reversal learning capacity, and to further explore the relationship between these post-cocaine cognitive deficits and drug-seeking during a 45 day period of cocaine abstinence. Towards this goal, this study utilized a novel combination of (1) drug self-administration with (2) an operant DMS/DNMS task and (3) (cue + context)-induced relapse that allowed for the sequential assessment of behavioral parameters in the same group of animals. The main finding of this study is that rats with a history of extended cocaine access show mild, but sustained deficits in working memory (DMS task), that do not predict the severity of drug-seeking. More pronounced cocaine effects might have been masked by the excessive incremental difficulty of the DMS task as discussed below.

The majority of studies in human cocaine addicts agree that cocaine use disorder is associated with prolonged general cognitive impairment, and that attention, impulsivity, and working memory are the most affected cognitive domains (for the recent review see [5]). Persistent post-cocaine deficits in working memory (assessed using a delayed alternation T-maze task) have also been detected in animal models utilizing cocaine self-administration [14]. In this study, we evaluated working memory/reversal learning in a novel experimental design that paired extended-access cocaine self-administration with an operant DMS/DNMS task. The advantages of automated tasks (such as the operant DMS/DNMS task) in comparison with traditional T-maze-based tasks include the ability to conduct a large number of trials per session without actively handling the animals or otherwise interrupting the ongoing behavior as well as the contribution to the standardization of protocols among laboratories [19]. Additionally, lesion studies indicate that intact PFC circuitry is required for the successful testing (high percentage of correct responses) in the DMS/DNMS task [17]. A critical parameter in utilizing the DMS/DNMS task to test cognitive performance is the level of difficulty (the length of delays) imposed during acquisition and the testing phase of this task. While rats tested on shorter delays might successfully and correctly complete a high number of trials, the working memory load might be too light to uncover cocaine-induced deficits. On the other hand, delays that are too long might be exceedingly difficult for rats to acquire, and instead lead to accumulation of incorrect responses or responding at chance (~ 50% correct). This pattern of responding was detected during DMS testing at the 20 s and 30 s delays in this study, indicating unsuccessful acquisition of the task. Furthermore, both groups performed below chance at each of the four delays {0 s, 10 s, 20 s, 30 s} in the DNMS task, suggesting that both groups failed to acquire the rule switch to the non-match condition. For this reason, it is appropriate to only consider groups differences at the delays {0 s and 10 s} in which the rats demonstrated above chance responding or learning of the task rules.

Performance at the 0 s delay is a measure of understanding the basic task rules of learning to press the same side lever during the match-to-sample condition. Both groups of rats performed the DMS task exceptionally well at the 0 s delay with an average of about 99%, which suggests that cocaine did not impair acquisition of the basic task rules. In addition, both groups did not differ in the number of days spent in training before the testing phase. This is consistent with other studies showing that cocaine history does not impair general cognitive abilities needed to perform delayed-match-to-sample T-maze tasks such as rule learning, learning rate and motivation for food incentives [13,14]. Performance at the 10 s delay was analyzed as a measure of working memory in the DMS task. There was not a significant difference between groups on the total percent correct at the 10 s delay taken as an average over the 5 day block of testing. However, compared to saline rats, the rats with a history of extended access-cocaine showed significant impairment at the 10 s delay on the last day of testing. This could indicate that cognitive deficits may become more pronounced further into abstinence. Additionally, this could suggest that rats in the saline group may improve over time while rats in the cocaine group may remain the same or even decline with repeated testing. However, no significant differences between groups in performance over time were detected in the current study and in two other studies [13,14].

The failure to perform above chance at longer delays (as detected in the current study), was due to experimental design (excessive incremental difficulty of the delays) as it affected both saline and cocaine rats. In other studies, working memory performance at ≥ 30 s delays has been above chance for both saline and cocaine groups, and significant impairments have been seen in the cocaine groups at these higher delays [13,14]. Specifically, it has been observed that compared to cocaine naïve rats, extended access cocaine rats exhibit deficits in working memory performance in a delayed-non-match to sample T-maze task when delays increase from 10 s to 70 s and 130 s [13]. Furthermore, extended access cocaine rats demonstrate significant working memory impairment in a delayed-alternation task compared to saline treated rats when the delay increased from 10 s to 30 s [14]. Similarly to our study, impairments seen in cocaine rats in these studies are not attributed to differences in learning rate or acquisition between the groups.

Although impairment was seen in the cocaine rats on the last day of testing at the 10 s delay, the aforementioned studies do not find differences between saline rats and cocaine rats at this delay. The discrepancy in our findings appears to be due to the difference in the types of tasks used. The other studies utilized T-maze tasks which require navigational exploration. It is possible that these comparable delays are more cognitively demanding in the operant delayed-match- to-sample task used in this study, since lever pressing for a food incentive is not as natural for a rat as exploring its surroundings and remembering navigational cues indicative of food. Support for this stems from the observation that normal and cocaine rats in the delayed-match to sample T maze task show greater than 85% correct responding at the 10 s delay whereas, saline and cocaine rats in this study showed a less than 60% correct responding at the same delay [13,14]. Thus, the operant delayed-match-to-sample task may require the use of shorter delays and more incremental training between higher delays. The choice of our delay set was driven by the use of these similar delays in the cocaine literature when investigating this domain of cognition. However, it is important to note that several studies have utilized this particular operant based DMS and DNMS tasks with more incremental delays of {0 s, 2 s, 4 s, 8 s, 12 s, 18 s, 24 s} when investigating cognitive performance in rodents [17,20]. These smaller increments between each delay interval allow the rat to be trained more appropriately for this type of task. Furthermore, this task has been shown to even be challenging for normal rats at the 18 s and 24 s delay with these rats performing with an accuracy of about 70% correct for each delay, but performance at the shorter delays are over 80% correct [17]. Here, even our saline rats exhibited less than 70% percent accuracy at just the 10 s delay indicating that more incremental training may be needed between higher delays. Thus, in our future experiments, we will adjust the task parameters by including these shorter delays with additional training at each delay set, and a maximum delay of 24 s.

What remains to be explored is the identity of neurobiological substrates with the PFC circuitry that could be responsible both for the post-cocaine hypofunction and post-cocaine cognitive deficits observed in humans (and some extent in animals). Recent evidence suggest that post-cocaine working memory decline coincides with structural changes within the PFC, such as decreased volume (humans, [21–23]), decreased dendritic branching and dendritic spine attrition (rats, [15]) and decline in a number of neurons and oligodendrocytes (rats, [13]). Out of an array of possible gene candidates responsible for the observed working memory deficits, our future studies will focus on the integrity of metabotropic glutamate receptor subtype 5 (mGlu5) signaling in the PFC. In support, mGlu5 receptors in the PFC as well as several other proteins regulating the magnitude of mGlu5 receptor signaling (such as RGS4) have been implicated in working memory function under physiological or pathological conditions [24–27].

In conclusion, the present study indicates that extended access to cocaine results in persistent working memory impairment at a moderate cognitive load (delay of 10 s). The failure to measure working memory performance at higher cognitive loads was due to an experimental design that allowed for increasing delays at a rate that was faster than the rate of acquisition. On the other hand, the current study suggests that an operant-based version of the DMS/DNMS task can be successfully used to measure cocaine-induced working memory/reversal learning deficits if sufficient incremental training precedes the testing phase. Future studies, using the updated DMS/DNMS design, will also re-evaluate whether the relationship between cognitive performance and cocaine-seeking can be detected using a more comprehensive cognitive dataset.
